# Assessment of Mancozeb Exposure, Absorbed Dose, and Oxidative Damage in Greenhouse Farmers

**DOI:** 10.3390/ijerph191710486

**Published:** 2022-08-23

**Authors:** Chiara Costa, Michele Teodoro, Federica Giambò, Stefania Catania, Silvia Vivarelli, Concettina Fenga

**Affiliations:** 1 Department of Clinical and Experimental Medicine, University of Messina, 98125 Messina, Italy; 2 Department of Biomedical and Dental Sciences, Morphological and Functional Imaging, Section of Occupational Medicine, University of Messina, 98125 Messina, Italy

**Keywords:** Mancozeb, ethylene thiourea (ETU), pesticides, occupational medicine, oxidative stress, risk assessment, advanced oxidation protein products (AOPPs)

## Abstract

Mancozeb (MNZ) is a fungicide commonly employed in many countries worldwide. This study assesses MNZ absorption dynamics in 19 greenhouse farmers, specifically following dermal exposure, aiming to verify the efficacy of both preventive actions and protective equipment. For data collection, a multi-assessment approach was used, which included a survey to record study population features. MNZ exposure was assessed through the indirect measurement of ethylene thiourea (ETU), widely employed as an MNZ biomarker. The ETU concentration was measured with the patch method, detecting environmental ETU trapped in filter paper pads, applied both on skin and working clothes, during the 8 h work shift. Urine and serum end-of-shift samples were also collected to measure ETU concentrations and well-known oxidative stress biomarkers, respectively, namely reactive oxygen metabolites (ROMs), advanced oxidation protein products (AOPPs), and biological antioxidant potential (BAP). It was observed that levels of ETU absorbed and ETU excreted were positively correlated. Additionally, working clothes effectively protected workers from MNZ exposure. Moreover, following stratification of the samples based on the specific working duty (i.e., preparation and spreading of MNZ and manipulation of MNZ-treated seedlings), it was found that the spreading group had higher ETU-related risk, despite lower chronic exposure levels. AOPP and ROM serum levels were higher in MNZ-exposed subjects compared with non-exposed controls, whereas BAP levels were significantly lower. Such results support an increase in the oxidative stress upon 8 h MNZ exposure at work. In particular, AOPP levels demonstrated a potential predictive role, as suggested by the contingency analysis results. Overall, this study, although conducted in a small group, confirms that ETU detection in pads, as well as in urine, might enable assessment of the risk associated with MNZ exposure in greenhouse workers. Additionally, the measurement of circulating oxidative stress biomarkers might help to stratify exposed workers based on their sensitivity to MNZ. Pivotally, the combination of both ETU measurement and biological monitoring might represent a novel valuable combined approach for risk assessment in farmhouse workers exposed to pesticides. In the future, these observations will help to implement effective preventive strategies in the workplace for workers at higher risk, including greenhouse farmers who are exposed to pesticides daily, as well as to clarify the occupational exposure levels to ETU.

## 1. Introduction

Mancozeb (Mn/Zn-ethylene-bis-dithiocarbamate, MNZ) is a widespread organometallic fungicide largely used in agriculture, as well as for diverse industrial applications [1]. MNZ derives from a combination of the two older dithiocarbamate (DTC) molecules, Maneb and Zineb, and, like most pesticides, it can be available as dust, liquid, dispersible granules, or wettable powder [2]. The production and market volumes of MNZ kept an increasing trend for several decades. This is because MNZ has a broad-spectrum efficacy towards a variety of plant diseases (including scab, rust, late blight, and leaf spot), and quite a low purchase price. From 2021, with a grace period which ended in January 2022, MNZ use as pesticide has been banned within the whole European Union (EU) due to the observed reproductive toxicity and endocrine disrupting properties [3,4,5]. Nevertheless, in several countries, MNZ is still largely employed in the agriculture sector [6].

The general population is passively exposed to fungicides through the environment, soil, water, consumption of contaminated wine and food (particularly tomatoes, citrus fruits, and potatoes), as well as cigarette smoking (because MNZ is largely used in tobacco cultivation) [7,8]. The professional exposure to MNZ especially occurs in the agriculture and farming industries, key sectors of MNZ production and application, either through the inhalation route or direct skin contact, although the dermal route represents the main route of exposure [9,10]. For such reasons, agricultural workers represent a subpopulation at higher risk of exposure to pesticides, including DTCs [11].

The fungicidal activity of DTCs is due to the release of ethylene-bis-isothiocyanate sulphide, which impairs enzyme functions by binding to metals and sulfhydryl protein groups [12]. DTCs are easily transformed in ethylene thiourea (ETU) within all environmental matrices, as well as through enzymatic transformations mediated by aquatic organisms, plants, and mammals. Additionally, ETU is employed as an accelerator in rubber vulcanization [13]. As a mechanism of action, ETU disrupts protein synthesis and metabolism by inactivating amino acidic sulfhydryl groups in the protein structure of fungal pathogens [14]. ETU is produced as a contaminant or as degradation product when fungicides are stored in the presence of moisture and oxygen. ETU may form in diluted suspensions of MNZ prepared for application on crops; hence, fair amounts of ETU may be present in the cultivar immediately after MNZ application. Additionally, ETU represents the primary catabolite of MNZ, formed after absorption at an approximate ratio of 1:2 (two molecules of ETU form one molecule of MNZ), and then excreted in urine [15]. Consequently, ETU is advocated as a biomarker of exposure to MNZ, providing human biomonitoring data, particularly in occupational settings [16].

Although DTCs are characterized by short persistence in the environment, causing mild acute toxicity upon exposure, MNZ and ETU are known to have additional long-term toxic effects of primary concern, due to their isothiocyanate skeletons, with molecular elements showing elevated binding capacity which may inhibit human enzymes, thereby affecting the interested biological systems and increasing the risk of endocrine disruption, cancer transformation, and neuronal damage [17,18,19]. 

Current scientific evidence in occupationally and environmentally exposed populations indicates that MNZ inhibits thyroid hormone (TH) receptor and impairs the hypothalamus–pituitary–thyroid axis. Additionally, the derivative ETU seems to act by reducing TH incretion through the inhibition of TH peroxidase. The consequent proliferative hyperstimulation due to thyroid-stimulating hormone action may induce thyroid cancer. This mechanism, observed in rodent studies, might suggest a possible carcinogenic effect also in exposed humans [20]. Nonetheless, the International Agency for Research on Cancer (IARC) classified MNZ as a Class 3 carcinogen given the limited evidence in humans, although teratogenic and carcinogenic effects have been observed in animal studies [5,21]. Additionally, DTC and/or ETU exposure has been associated with neurodevelopmental damage due to the well-known crucial role of thyroid function in brain development [9,22]. Recent studies suggest possible disrupted neurobehavioral outcomes and neurotoxicity with Parkinson-like neuronal damage upon MNZ exposure [23,24]. 

There is evidence from epidemiology studies, as well as from experimental data, that MNZ exposure should be considered a risk factor for developmental and reproductive dysfunction in humans [25,26,27]. MNZ and ETU can cross the placental barrier, directly affecting reproduction at preconception, pregnancy, and birth stages [28]. A number of in vitro studies report that ETU reactivity is associated with direct DNA damage [29,30,31], nitrosamine formation [32], apoptosis induction [33], and the altered expression of genes involved in oxidative stress response [34,35], overall causing the gradual accumulation of damage to intracellular macromolecules exerted by reactive oxygen species (ROS) [36,37]. The consequent oxidative stress might be pivotal in the pathogenesis of thyroid, neural, and immune toxicity [38]. 

Despite the scientific progress made in understanding the molecular mechanisms linked to MNZ toxic effects, limited research has been performed on risk assessments based on real-life occupational sceneries, including the identification of the determinants of exposure typical of pesticide manipulation in the field. During mixing and dispersion procedures in the workplace, MNZ absorption occurs mainly through cutaneous routes. Dermal assessment is considered relevant for the evaluation of pesticide exposure, especially in greenhouses, where the temperature is often very high. In fact, both sweating and increased skin permeability boost the absorption of substances which would not otherwise overcome the dermal barrier under normal conditions [4,39].

Based on these premises, this study aims to fill the gap in knowledge regarding the evaluation of MNZ absorption dynamics in greenhouse farmers. The primary goal was to verify the efficacy of both preventive actions and protective equipment. For this purpose, we developed a tiered approach, coupling a lifestyle and exposure questionnaire with the measurement of biomarkers which might be correlated with MNZ occupational exposure [40]. Firstly, ETU dermal exposure and urine excretion were determined, thus allowing the evaluation of the actual absorbed dose and the risk associated. Moreover, based on the importance of oxidative stress as the main mechanism of toxic effect for many types of pesticides including DTCs [41], our investigation included the measurement of relevant oxidation parameters in farmers professionally exposed to MNZ. In particular, blood concentrations of reactive oxygen metabolites (ROMs), advanced oxidation protein products (AOPPs), and biological antioxidant potential (BAP) were assessed as potential circulating biomarkers of MNZ toxicity [42]. The combination of ETU assessment with biological monitoring might represent a novel valuable approach for improved risk evaluations in workers exposed to pesticides. Additionally, the possible identification of predictive oxidative stress biomarkers might be suggestive of relevant exposure pathways to be explored further.

## 2. Materials and Methods

### 2.1. Study Design

This study was carried out in the greenhouse of a farm located in Eastern Sicily, Italy, in March 2019 (with average outdoor temperature ranging from 5 °C to 20 °C), before the MNZ ban. Specifically, tomato, watermelon, melon, cucumber, and aubergine were grown in the greenhouse. The greenhouse respected EU standard regulations (i.e., CSN EN 13031-1), with a constantly controlled internal microclimate. In particular, inside the greenhouse, soil and air temperature, relative humidity, carbon dioxide (CO_2_) concentration, light, and soil moisture were maintained at the optimal level (i.e., average internal temperature 20 °C, mean daily solar radiation 2–2.3 kW h/m^2^, internal humidity 60–90%, internal CO_2_ concentration 700–900 μmol/mol). Additionally, both outside parameters (e.g., external temperature, solar radiation, wind) and equipment (pipe temperature, vents, and curtains) were constantly monitored.

According to current Italian legislation, greenhouse workers were included in a compulsory medical surveillance program. On that occasion, they were invited to participate in the investigation activity without any reward. All the workers who voluntarily accepted to participate to the study provided signed informed consent. This study was carried out in accordance with the Declaration of Helsinki’s ethical standards. Being part of the mandatory occupational health surveillance, the study needed no formal approval from the local ethics committee.

The plants in the greenhouse were treated with a commercial powder formulation known as FORUM MZ WG ^®^ (60% MNZ, BASF spa, Italy), solubilized before spreading, into a tank filled with cold water at final concentration of 2.5 g/L with an averaged concentration of 1.5 g/L of MNZ in the solution. The mean area of the sprayed field was 500 m^2^. MNZ solution was prepared immediately before use. The pesticide was dispersed with spray lances, using traditional handheld spray application techniques with standard spray guns. The operator, walking forwards and backwards, always kept the spray lance forward. The height of the nozzles was about 50 cm above the ground. Overall, the preparation time of the pesticide solution and its sprinkling over the field lasted about 8 h (covering the entire work shift). The day following MNZ spreading over the plant field, personnel involved in the seedling planting worked for the entire work shift (8 h) manipulating MNZ-treated seedlings.

**Figure 1:**
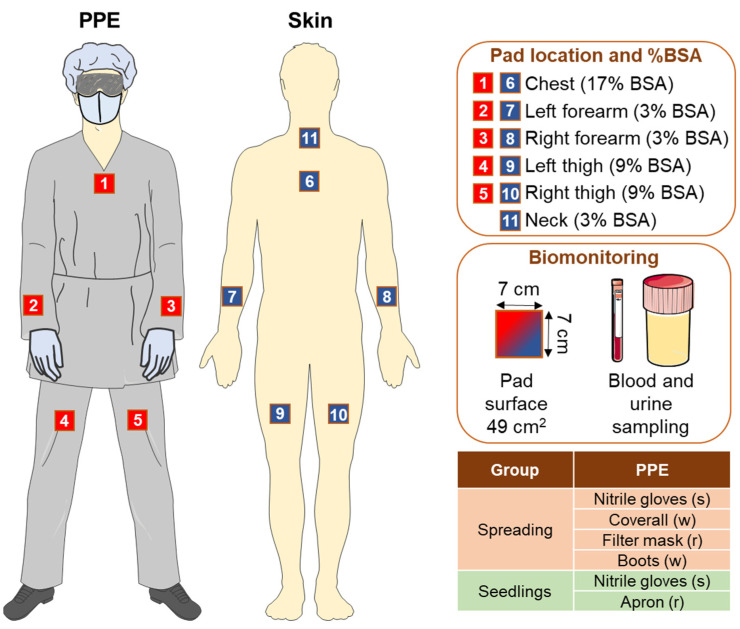
Features of the biological assessment. On the left are reported the body locations of the 11 pads used in the patch-based monitoring of ETU levels, applied on both working clothes and skin. On the right, for each pad is indicated the location and the corresponding represented percentage of body surface area (BSA). Additionally, the biomonitoring procedures (i.e., pad surface, end-of-shift blood, and urine sampling), and the personal protective equipment (PPE) used are indicated. Abbreviations: s, single use; w, washable; r, reusable.

In the field of occupational medicine, risk assessments are performed with specific environmental and biological measurements with the aim of objectifying worker exposure levels and then comparing them with reference values. In particular, the dose of ETU (main MNZ metabolite) absorbed via the dermal route was assessed with the use of specific square pads made of cellulose Whatman filter paper (Merck KGaA, Darmstadt, Germany), with total effective surface of 49 cm^2^. The so-called “patching” method employs pads either directly attached to the skin or to the protective clothes of workers. A total of 11 pads were used for the passive monitoring: 5 pads were attached to the work clothes used during the work shift (red numbered squares, Figure 1), and 6 pads were directly applied to the skin (blue numbered squares, Figure 1), according to [43].

In particular, 5 pads were positioned on the chest, thighs, and forearms, both on clothes and on skin. A single pad was attached at the level of the skin of the neck with no protective clothes above. In order to perform the monitoring, pads were worn by the farmers for the whole work shift, lasting 8 h. Pads on the clothes estimated the amount of ETU that could be potentially assimilated by the subject. Pads attached under the clothes (to the skin) estimated the amount of ETU that could actually be absorbed through the skin.

### 2.2. Subjects Enrolled and Timeline of the Study

Workers involved in the study voluntarily joined and signed the informed consent at the enrolment. The assessments were performed during a normal workday, without disrupting the usual working habits. A total of 19 farmers were enrolled in the study, 2 of which (males) were involved in the preparation and spreading of MNZ over the field, while the remaining 17 workers (females) were employed in the planting of MNZ-treated seedlings. There were 19 study participants in total, aged between 21 and 69 years (Table 1). All the subjects participating in the study were Caucasian.

The body mass index (BMI) was calculated as the weight (kg) divided by the square of height in meters (m^2^). The body surface area (BSA) was calculated according to the Mosteller formula:

(1) $$\mathrm{BSA}\;\left(m^{2}\right)=\sqrt{\frac{\mathrm{height}\;\left(\mathrm{cm}\right)\times \mathrm{weight}\;\left(\mathrm{kg}\right)}{3600}}$$

The percentages of body surface represented by each pad were calculated, normalizing each BSA for the specific body part percentage (Figure 1). For blood biomarker comparisons, a control group of 21 indoor workers was also identified, which was composed of subjects not exposed to phytosanitary conditions in the workplace, house, or environment, and with socio-demographic characteristics comparable to the greenhouse workers.

The study was carried out in 3 days. During the first day (pre-sampling), the 19 enrolled workers filled a survey to collect personal data and information related to the job performed, phytosanitary used, type of cultures, working time and frequency of pesticide treatments over a year, and information on the hygiene and health procedures and the personal protective equipment (PPE) used. Information regarding any other exposure to chemical agents, symptoms possibly related to MNZ exposure occurring in the last 12 months, smoking habits, eating habits, occurring diseases, and the use of medications were also recorded (Table 2). All participants declared to have received adequate information regarding risk exposure and safety procedures and to follow instructions during work activity.

During the second day, the 2 workers involved in the MNZ solution preparation and spreading were monitored with pads (8 h working day). Pads were attached to both skin and protective clothes from the start to the end of the workday. Additionally, end-shift urine and blood samples were collected. The third day, the 17 operators involved in the processing of seedlings, which were sprayed with MNZ the day before, were monitored with the same sampling procedures (8 h working day monitoring; pad applications lasted for the entire work shift and end-shift urine and blood sampling, Figure 1).

### 2.3. Pads, Blood and Urine Sampling, Transport and Conservation Procedures

Pads were applied before the work shift started, including before the operation of pesticide weighing and mixing, and removed at the end of the work shift. Once removed, the pads were protected from light with aluminium foil to avoid ETU degradation [44,45]; then, they were transported inside refrigerated bags and stored at 4 °C until further analysis. At the end of the work shift, both blood and urine samples were collected from each participant (Figure 1). Blood samples were collected in a serum collection tube. These samples were kept refrigerated during transport, then centrifuged at 1000× *g* rpm, and separated serum was harvested and stored in a sterile clean tube at −20 °C until further analysis. Urine samples were collected in plastic sterile single-use containers protected from light with aluminium foil, and stored at −20 °C until further process. Sample volumes and creatinine reference levels were determined in all the collected samples to adjust for the urinary dilution. Urinary creatinine was analyzed with a routine clinical method.

### 2.4. Chemicals

MNZ (CAS number 8018-01-7; chemical formula C_8_H_12_MnN_4_S_8_Zn) was purchased from BASF spa, Italy. Internal standard (IS) deuterated Ethylene thiourea, [^2^H_4_]-ETU, and ETU (CAS number 96-45-7; chemical formula C_3_H_6_N_2_S); Phosphate-buffered saline (PBS) Bio-Performance Certified, pH 7.4; Acetic acid (CAS number 64-19-7; chemical formula CH_3_COOH); Chloramine-T hydrate ≥95% (CAS number 149358-73-6; chemical formula C_7_H_7_ClNNaO_2_S); Trichloroacetic acid ACS reagent ≥99.0% (CAS number 76-03-9; chemical formula CCl_3_COOH); 1,1,3,3-Tetraethoxypropane ≥96% (CAS number 122-31-6; chemical formula C_11_H_24_O_4_) were purchased from Sigma-Aldrich (Munich, Germany). Sodium hydroxide (CAS number 1310-73-2; chemical formula NaOH) tablet for analysis ACS AnalaR NORMAPUR and Formic acid ≥99% (CAS number 64-18-6; chemical formula HCOOH) LC-MS grade, HiPerSolv CHROMANORM, were both purchased from VWR International (Radnor, PA, USA). Hydrochloric acid >37% (CAS number 7647-01-0; chemical formula HCl) was purchased from Honeywell Fluka (Charlotte, NC, USA). n-Butanol (CAS number 71-36-3; chemical formula C_4_H_9_OH) EMSURE was purchased from Supelco Inc. (Bellefonte, PA, USA). Upon preparation, stock solutions of internal standards [^2^H_4_]-ETU and ETU (prepared at 1 mg/mL in acetonitrile) and diluted standards were stored at −18 °C.

### 2.5. Pads and Urine Sample Processing and Calibration Analyses

Filter paper pads were placed in 20 mL test tubes with 6 mL of distilled water. Tubes were placed in a sonicating bath for 60 min without heating and then mixed for 20 min with the use of a mechanical shaker. The aqueous solution was acidified with 20 µL of 50 M HCl and filtered. The acidified solutions were analyzed through chromatography according to Kurttio method [46]. Urine samples were thawed and processed following Ekman method for subsequent analysis [47].

For the calibration curve, 400 µL of water extracted from pads and 400 µL of urine were spiked with 50 µL of standard solution and 50 µL of IS solution. The urinary calibration curve was developed using ETU-free urine from healthy volunteers. The same procedure was used to prepare quality control samples. The analysis of both pad and urine ETU was conducted using high-pressure liquid chromatography coupled with a triple-quadrupole mass spectrometer (LC-MS-8030 Shimadzu, Tokyo, Japan). A detector with an electrospray ionization interface (ESI), operating in positive ion mode, was used to acquire mass spectra (MS_1_), product ion spectra (MS_2_), and for quantitative analyses. ESI voltage settings are reported in Supplementary Table S1, whereas MS_1_ and MS_2_ are reported in Supplementary Table S2. The limit of detection (LOD) and limit of quantification (LOQ) were calculated through a six-point calibration curve, obtained with a linear range from 0.25 to 200 μg/L (Supplementary Table S3). 

Linear regression and square of the correlation coefficient (r^2^) were used to measure the fitness of the curve, which was >0.998. The precision of the method for each concentration is expressed as a coefficient of variation (CV) by calculating the standard deviation (SD) as a percentage of the mean calculated concentration, whereas the accuracy of the assay was determined by expressing the mean calculated concentration as a percentage of the added concentration. Intra-day and inter-day titration results are reported in Supplementary Tables S4 and S5, respectively.

### 2.6. Serum Biomarkers for the Measurement of Oxidative Stress

The overall oxidative status was evaluated measuring the levels of ROM present in serum samples. The automated spectrophotometric method used was the d-ROM test (Diacron srl, Grosseto, Italy), according to Hirose et al. [48]. ROM measurements were expressed as Carr Units (CARR-U). The plasma antioxidant barrier was evaluated through the detection of BAP serum levels with a commercial kit (Diacron srl, Italy). BAP levels were assessed using ferric chloride (FeCl_3_) as a sensor, and measuring the capability of the serum sample to reduce an iron Fe^3+^ ion to an iron Fe^2+^ ion. The 505 nm absorbance was measured through spectrophotometric analysis and the values obtained were calculated as µmol/L (μM), in comparison with a calibration curve, according to [48,49]. AOPP serum levels were measured using a spectrophotometric method previously described by Costa et al. [50]. Briefly, 200 µL of serum was diluted 1:5 with 0.1 mol/L pH 7.4 PBS; subsequently, 20 µL of 14 M acetic acid and 10 µL of 1.16 M potassium iodide were added. Chloramine-T solution (0–128 μM) was used for creating the calibration curve. Absorbance was measured at 340 nm using the same instrumentation as reported above and the AOPP concentration was expressed in chloramine-T units (µmol/L, μM). All spectrophotometric measurements were performed using a Sinergy HT microplate reader (Bio-tek, now part of Agilent, Santa Clara, CA, USA).

### 2.7. Data Processing and Exposure Assessments

From concentrations of ETU (mg/L) extracted from individual pad samples, the absolute amount in the original sample was calculated and expressed in total µg. From the total µg of ETU, the quantity of ETU normalized for the area of each pad, expressed as ng/cm^2^, was calculated. The exposure level calculation considered pivotal time parameters for each worker enrolled, which are the working seniority (years, Y) and the exposure duration (days per year, D). The chronic exposure level (CEL), expressed in arbitrary units (au), was calculated applying the formula:

(2) $$\mathrm{CEL}=\log_{10}\left[\left(\frac{Y\times D}{\mathrm{age}-18}\right)+1\right]$$

For each exposed subject, the age at the time of the assessment has been recorded as Y, corresponding to the number of years of occupational exposure to MNZ, and D, corresponding to the number of days per year of MNZ utilization (8 h day shift), thereby obtaining the corresponding CEL, according to [51].

The potential skin exposure (PSE) of the entire body to ETU, which is the amount of ETU that reaches the clothes of the individual, was calculated according to Mandic-Rajcevic et al. [52]. It corresponds to the sum of each regional exposure measured by pads attached to the clothes. In detail, considering that the surface of each pad is 0.49 dm^2^:

(3) $${\mathrm{PSE}}_{\mathrm{body}}\;\left(\mathrm{mg}\right)=\overset{5}{\underset{\mathrm{pad}=1}{\sum }}{\mathrm{ETU}}_{\mathrm{pad}}\;\left(\frac{\mathrm{mg}}{{\mathrm{dm}}^{2}}\right)\times {\mathrm{body}\;\mathrm{area}\;\mathrm{represented}}_{\mathrm{pad}}\;\left({\mathrm{dm}}^{2}\right)\;$$

where the body area represented by each pad is calculated as the BSA normalized for the specific surface area corresponding to the pad (Figure 1).

Analogously, the actual skin exposure (ASE) of the entire body to ETU, which represents the amount of ETU reaching the skin (hence, available for absorption), was calculated according to Mandic-Rajcevic et al. [52], as the sum of each regional exposure measured in the skin pads multiplied for the body area represented by each single pad, as follows:

(4) $${\mathrm{ASE}}_{\mathrm{body}}\;\left(\mathrm{mg}\right)=\overset{11}{\underset{\mathrm{pad}=6}{\sum }}{\mathrm{ETU}}_{\mathrm{pad}}\;\left(\frac{\mathrm{mg}}{{\mathrm{dm}}^{2}}\right)\times {\mathrm{body}\;\mathrm{area}\;\mathrm{represented}}_{\mathrm{pad}}\;\left({\mathrm{dm}}^{2}\right)\;$$

Through the comparison of PSE and ASE values, for each study subject it was possible to calculate the protection percentage, according to the following formula:

(5) $$\mathrm{Protection}\;\mathrm{percentage}=\frac{\mathrm{PSE}}{\mathrm{PSE}+\mathrm{ASE}}\times 100$$

Considering that, according to [53], the ETU dermal absorption coefficient is 0.26, the absorbed dose of ETU via dermal route per kilogram of body weight may be calculated as follows:

(6) $${\mathrm{ETU}\;\mathrm{absorbed}}_{\mathrm{kg}\;\mathrm{bw}}=\frac{\mathrm{ASE}\times {\mathrm{Dermal}\;\mathrm{Absorption}\;\mathrm{Coefficient}}_{\mathrm{ETU}}}{\mathrm{Body}\;\mathrm{Weight}}$$

Finally, considering that, according to [4], the acceptable operator exposure level (AOEL) for ETU is 0.002 mg/kg, the percentage risk associated with ETU dermal exposure may be calculated as:

(7) $$\mathrm{Risk}\;\mathrm{ETU}\;\mathrm{dermal}\;\mathrm{exposure}=\frac{{\mathrm{ETU}\;\mathrm{Absorbed}}_{\mathrm{kg}\;\mathrm{bw}}}{{\mathrm{AOEL}}_{\mathrm{ETU}}}\times 100$$

Regarding ETU levels measured in urine, each raw value was normalized for the urine total volume and the creatinine reference levels, to finally obtain the levels of creatinine of ETU in each urine sample (µg/g). According to Mandic-Rajcevic et al. [53], the risk associated with the ETU measured in urine, which can be correlated to both the catabolism of absorbed MNZ and environmentally formed ETU from MNZ degradation in the field, may be calculated as follows:

(8) $$\mathrm{Risk}\;\mathrm{ETU}\;\mathrm{in}\;\mathrm{urine}=\frac{{\mathrm{ETU}}_{\mathrm{urine}}\;\left(\mathrm{mg}/g\;\mathrm{creatinine}\right)}{\mathrm{Body}\;\mathrm{Weight}\;\times {\;\mathrm{ADI}}_{\mathrm{ETU}}}$$

where the acceptable daily intake (ADI) of ETU is estimated to be 0.004 mg/kg bw [53]. 

### 2.8. Statistical Analyses

Data processing and statistical analyses were performed using GraphPad Prism version 9.0 for Windows (GraphPad Software, La Jolla, CA, USA). For each variable of interest, Shapiro–Wilk normality tests were run. The results are presented as medians with indications of minimum and maximum values. For normally distributed data, single-parameter comparisons between two groups were conducted using two-tailed unpaired Student’s t-tests, whereas single-parameter comparisons between three or more groups were performed using one-way analysis of variance (ANOVA) with Tukey’s multiple comparison test. For non-normally distributed data, non-parametric tests were used, and single-parameter comparisons between two groups were conducted using Mann–Whitney tests, whereas single parameter comparisons between three or more groups were performed using Friedman tests, correcting for multiple comparisons using the suggested Dunn’s test. Depending on the distribution of data, correlations between different parameters were evaluated by calculating the Pearson’s or Spearman’s correlation r coefficients (used with either normal or non-normal data distributions, respectively). Contingency analyses were performed using Fisher’s exact test (odds ratio calculation with Baptista–Pike post-test). For all statistical analyses, differences were considered significant with *p*-values < 0.05; with * *p* < 0.05; ** *p* < 0.01; *** *p* < 0.001; **** *p* < 0.0001.

## 3. Results

### 3.1. Chronic Exposure Levels Were Significantly Higher in the Seedling Group Compared with the Spreading Group

A total of 19 healthy workers voluntarily participated to this study (Figure 1). For simplicity, personnel involved in the preparation and spreading of MNZ were called “spreading”, while individuals involved in the processing of MNZ-treated seedlings were called “seedlings”. The main relevant features of the individuals enrolled in this study are summarized in Table 1 and Table 2.

**Figure 2:**
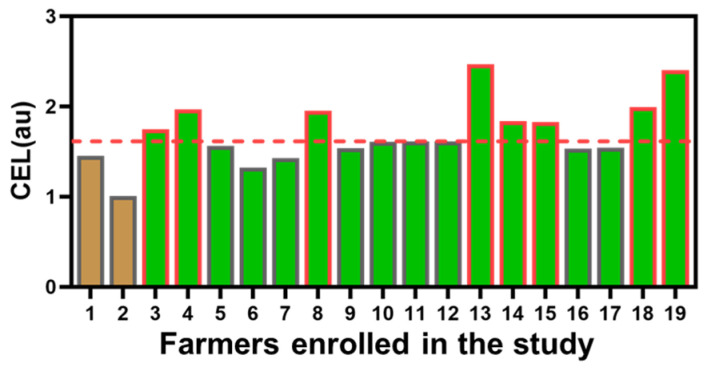
Chronic exposure levels (CELs) in MNZ-exposed workers. Numbers from 1 to 19 in the x axis univocally identify each subject enrolled in the study. Brown bars indicate CEL values calculated for spreading workers, green bars indicate CEL values calculated for seedling workers. Red dotted line at 1.61 au indicates the cut-off median value. Bars with red borders indicate samples with CEL au values above the cut-off limit.

As reported in Table 1, the measured CEL was significantly higher in the seedling group compared with the spreading group (median of 1.61 versus 1.23 au, respectively, *p* = 0.039). In the seedling group, 8 out of 17 individuals (47%) showed CEL values above the calculated median (median of 1.61 au, above-median values as red border columns, Figure 2).

This result could be explained with the different exposure durations of the two groups, being significantly reduced for the spreading group compared with the seedlings group (with 45 declared working days per year for the spreading group, versus 220 working days per year for the seedling group, *p* < 0.0001).

Regarding the lifestyle habits, as shown in Table 2, 58% of the enrolled workers were non-smokers, 100% did not consume alcohol, 79% declared to drink coffee or tea during the day, 100% of them consumed vegetables daily, and 79% consumed fruit regularly. Only 26% declared symptoms possibly related to exposure to pesticides in the last 12 months (skin irritation), 32% mentioned occurring pathologies, and 47% declared to have assumed medications in the last 30 days before the survey. 

### 3.2. Exposure Assessment in Pads Shows Significantly Higher ETU Levels in the Spreading Group

**Figure 3:**
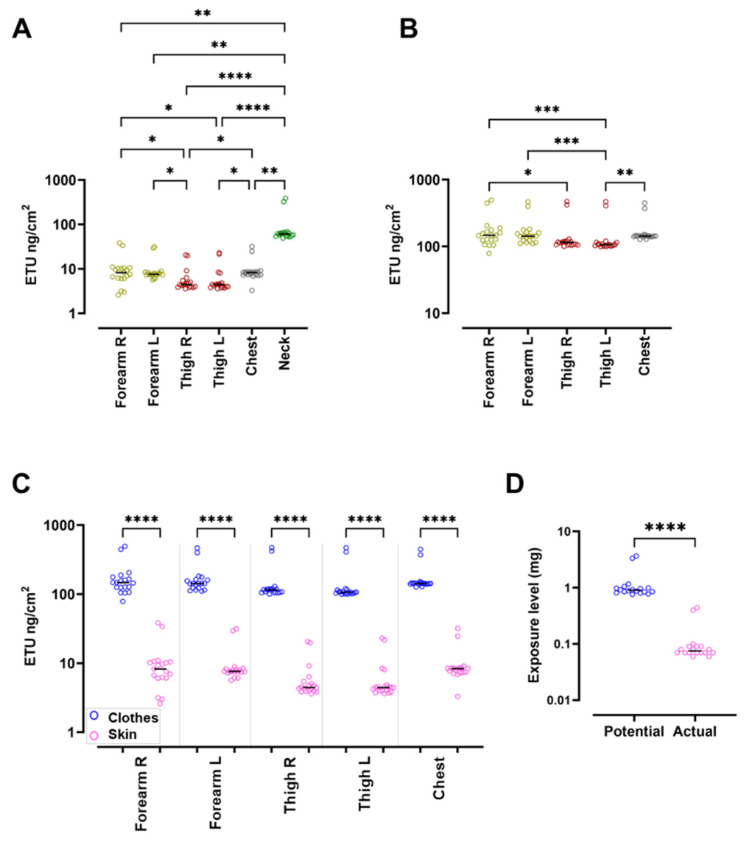
Levels of ETU measured in the pads located on the skin and on the clothes. (**A**) Levels of ETU expressed in ng/cm^2^ detected in skin pads. (**B**) Levels of ETU expressed in ng/cm^2^ detected in clothes pads. (**C**) Levels of ETU expressed in ng/cm^2^ detected in pads of same body location of both skin and clothes. (**D**) Box plot showing the potential and actual exposure values (expressed in mg) considering all the enrolled subjects. (**A**,**B**) Yellow circles, forearm; red circles, thigh; grey circles, chest; green circles, neck. (**C**,**D**) Blue circles, pads applied on clothes; pink circles, pads applied on skin. (**A**–**C**) Friedman test multiple comparisons with Dunn’s post-test. (**D**) Wilcoxon test matched pairs. Data are reported as dot plots with median values. * *p* < 0.05; ** *p* < 0.01; *** *p* < 0.001; **** *p* < 0.0001. Where no asterisk is reported, the comparison between groups is not significant.

Pads represent a valuable and widely used tool for environmental and biological monitoring for compounds with relevant skin exposure and bioavailability [52]. Figure 3 shows the levels of ETU (ng/cm^2^) measured in all pads and the relative statical differences. 

Levels of ETU detected in skin pads were significantly higher in the neck pad, with a median value of 60.9 ng/cm^2^ (maximum 387.2 ng/cm^2^ and minimum 49.29 ng/cm^2^); this could be correlated to the fact that the neck body area was directly exposed to the environment with no protective clothes above (Figure 3A). Thigh ETU levels were the lowest measured, both in clothes and skin pads (Figure 3A,B). Interestingly, ETU levels detected in skin pads were significantly lower than in the corresponding area assessed with clothes pads (Figure 3C).

As shown in Table 3, from the measured ETU levels detected in pads, it was possible to calculate the PSE, whose median value was 0.90 mg considering the whole population (maximum 3.65 and minimum 0.76 mg), 3.49 mg considering the spreading group (maximum 3.65 and minimum 3.33 mg), and 0.87 mg considering the seedling group (maximum 1.16 and minimum 0.76 mg). Hence, there was a significant difference between the potential ETU exposure of the two groups of workers, being higher in the spreading group (*p* < 0.0001). In line with this result, the calculated ASE was significantly higher in the spreading group compared with the seedling group (with calculated medians of 0.42 and 0.07 mg, respectively, *p* < 0.0001, Table 3).

Considering all the enrolled subjects, the actual ETU exposure levels were significantly lower compared with the potential levels of about tenfold greater, with median values of 0.90 mg (minimum 0.76 mg and maximum 3.65 mg) and 0.07 mg (minimum 0.06 mg and maximum 0.44 mg, *p* < 0.0001, Figure 3D), respectively. The calculated protection percentage was 92% for seedling workers compared with 89% in spreading workers. Correspondingly, the median ETU absorbed was 0.30 and 1.33 µg/kg bw, respectively, whereas the risk calculation obtained with pads assessment was 15% versus 67% for the seedling and the spreading groups, respectively (Table 3).

### 3.3. ETU Excreted in Urine Was Significantly Higher in the Spreading Group

Regarding ETU detected in urine samples, as described in Table 3, the median ETU value measured considering the entire population was 8 µg/g of creatinine. The values were significantly different between spreading and seedling groups, with median values of 171.6 and 6.3 µg/g of creatinine, respectively (*p* < 0.0001). In 74% of the individuals enrolled, ETU values in urine were greater than 2.5 µg/g of creatinine, which is considered the suggested cutoff reference in healthy subjects [54]. As reported in Table 3, the corresponding risk calculated based on the urine ETU assessment was 51.8% (minimum 43.6% and maximum 59.9%) in the spreading group and 2.5% (minimum 0.1% and maximum 30.5%) in the seedling group (*p* < 0.001), in line with the risk calculated with pad ETU measurements.

**Figure 4:**
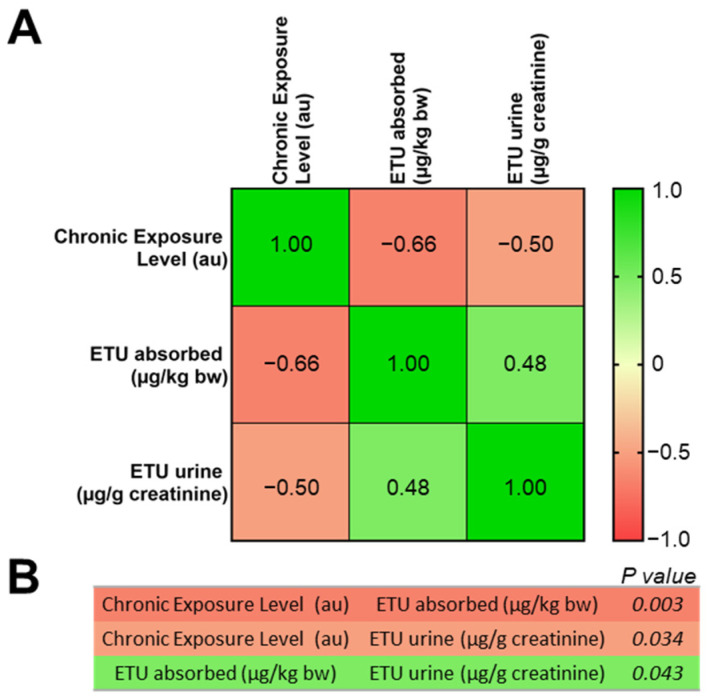
ETU exposure correlation matrix in greenhouse workers. (**A**) Spearman’s correlation matrix between chronic exposure levels (arbitrary units, au), ETU absorbed (µg/kg bw) and ETU measured in urine (µg/kg creatinine). Within each square, the corresponding r Spearman value is indicated, with associated color-code: green meaning a positive correlation, and red meaning a negative correlation. (**B**) *p* values corresponding to correlation values.

The correlation matrix in Figure 4 shows that the CEL calculated, which is based on the working seniority and the annual working days, is inversely correlated with both ETU absorbed (from pads assessment) and ETU levels detected in urine (Spearman’s correlation values of −0.66 and −0.50, respectively). In line with that observation, the values of ETU absorbed and ETU detected in urine are positively correlated (Spearman’s correlation value of 0.48).

### 3.4. Oxidative Stress Marker Levels Are Associated with the Risk Evaluation from Pads and Urine Monitoring

**Figure 5:**
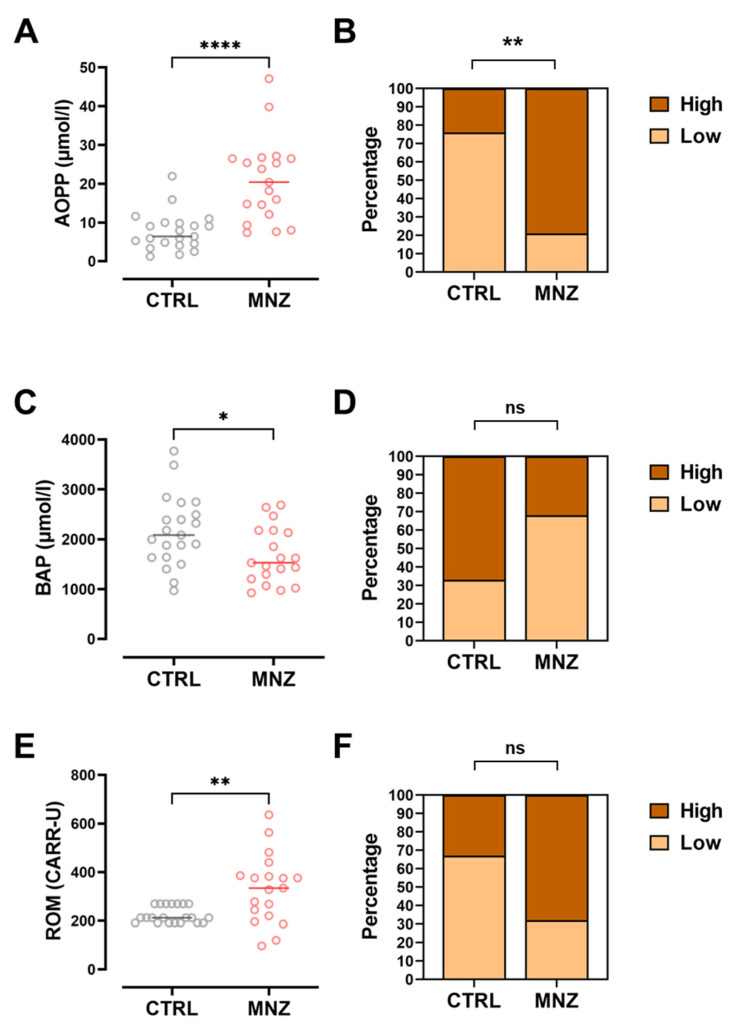
Oxidative stress serum biomarker assessment in MNZ-exposed greenhouse workers compared with non-exposed control population. (**A**) AOPP levels (expressed in µmol/L) in control non-exposed (CTRL) and MNZ-exposed (MNZ) workers. (**B**) Contingency analysis for AOPP expression (percentage of samples with high versus low marker expression, cutoff median value, 9.99 µmol/L). (**C**) BAP levels (expressed in µmol/L) in CTRL and MNZ workers. (**D**) Contingency analysis for BAP expression (percentage of samples with high versus low marker expression, cutoff value the median, 1881.54 µmol/L). (**E**) ROM levels (expressed in CARR-U) in CTRL and MNZ workers. (**F**) Contingency analysis for ROM expression (percentage of samples with high versus low marker expression, cutoff value the median, 256.50 CARR-U). (**A**,**C**,**E**) Grey circles, controls non-exposed, CTRL; red circles, mancozeb-exposed, MNZ. In (**A**,**C**,**E**) data are reported as dot plots with median values. For group comparisons, either Mann–Whitney tests or unpaired t tests were used, according to the distribution of data. * *p* < 0.05; ** *p* < 0.01; **** *p* < 0.0001; ns = not significant.

In order to evaluate the oxidative stress in the farmers enrolled in this study, AOPP, BAP, and ROM serum levels were compared with those detected in serum samples from 21 non-exposed indoor workers. The results shown in Figure 5A,C,E demonstrate that MNZ exposure is associated with a significant increase in all the measured oxidative stress biomarkers.

Specifically, in MNZ-exposed individuals and non-exposed controls, median AOPP serum levels were 20.4 and 6.4 µmol/L, respectively (*p* < 0.0001), and median ROM serum levels were 334 and 212 CARR-U, respectively (*p* = 0.0018). In line with these results, BAP serum levels were 1529 and 2084 µmol/L (*p* = 0.020), respectively, in MNZ-exposed individuals and non-exposed controls. To evaluate the predictive value of such differences, contingency analyses with Fisher exact test were conducted, demonstrating that specifically AOPP levels measurement in serum might additionally have a significant predictive value (Figure 5B,D,F; *p* = 0.0012).

## 4. Discussion

Safety measures required by law in most countries should provide adequate health protection for occupationally exposed subjects, given that they arise from rigorous risk assessment processes. A number of variables may contribute to determining both potential and actual exposure profiles of farmers to pesticides. For example, crop type, cultivation techniques, pesticide dispersion methods, technological standard achieved, maintenance of spreading tools, and climate issues represent critical factors which may all contribute to the overall exposure. 

In particular, the toxic effects of MNZ exposure in 19 greenhouse workers have been evaluated in this study. To this aim, a combined approach was employed. Firstly, the current health status and population features were evaluated with a pre-enrolment survey. Secondly, for each enrolled worker, exposure assessments were performed, with the application of the “patches” monitoring methodology, as well as the concurrent sampling of urine and blood at the end of the 8 h work shift for ETU and oxidative stress parameter evaluation (Figure 1). The patches technique had a main goal of measuring the pad-trapped ETU concentration, which is the main metabolite formed from MNZ either in the field after spreading or in the body upon absorption and subsequent metabolism; hence, it is a widely used indicator of MNZ exposure [16]. Moreover, the ETU excretion following 8 h MNZ exposure has been also monitored through urine level measurements. Finally, blood levels of oxidative biomarkers (i.e., AOPP, BAP, and ROM) were evaluated, as potential indicators of MNZ toxicity.

The initial evidence suggested by the results obtained here indicates that different work activities might be correlated with diverse doses of MNZ to which a worker comes into contact. All the farmers worked in the same greenhouse-controlled environment, but whereas 2 male individuals prepared and spread MNZ in the field (exposure time of 8 h, corresponding to the entire working day), the remaining 17 female subjects were engaged in manipulating the MNZ-treated seedlings for the whole 8 h shift. As showed in Table 3, pad-based assessment evidenced an overall PSE of 0.90 mg, significantly different when samples were split in the two groups. In fact, despite the small number, spreading male workers showed a median PSE of 3.49 mg, four times higher than what measured in female seedling group (which was a median value of 0.87 mg). Coherently, the ASE, with an overall measured median of 0.07 mg, was six times higher in the spreading group than in the seedling group (0.42 mg versus 0.07 mg median values). 

The actual median ETU absorbed was 0.31 µg/kg bw considering all the farmers enrolled. When samples were stratified according to the job, spreading workers showed a median value of 1.33 µg/kg bw, fourfold higher than the seedling workers (median of 0.30 µg/kg bw). The ETU exposure recorded with the patch system enabled calculation of the risk associated, which was a median of 15% when considering all samples, but 4.5-fold higher in the spreading group than the seedling group, when samples were stratified based on work duties (15% versus 67% median values, respectively).

The excreted urine ETU levels measured mirrored what was observed for the ETU absorbed values, with an overall measured median ETU urine concentration of 8 µg/g creatinine. The concentration values were significantly different when subjects were split in spreading and seedlings subgroups, with measured median ETU urine concentration 27 times higher in the spreading group compared with the seedling group (medians of 171.6 versus 6.3 µg/g creatinine). Importantly, the Spearman’s correlation analysis confirmed a significantly positive correlation between ETU absorbed and ETU excreted, with an r value of 0.48 (*p* = 0.043).

Through a calculation which considered the exposure time (in terms of both years of seniority and days of exposure per single year), it was possible to calculate the CEL, which is independent from the ETU measurements obtained with pads and urine sampling. Interestingly the median CEL calculated including the entire study population was 1.61 au; however, when subjects were stratified based on specific job duties, there was a slight but significant difference, this time in favor of the spreading group, where the median values were below the overall median (1.23 au, Table 1). The seedling group showed 1.3-fold higher median values of CEL (1.61 au), with 47% of the samples showing au values above the calculated median, therefore considered significantly chronically exposed according to this parameter (Figure 2) [51]. Interestingly, two out of three seedling group females with declared thyroid issues showed CEL values above the established threshold (i.e., 1.84 and 2.40 au), and both subjects had long working seniority (12 and 16 years, respectively). Hence, according to the CEL analysis, the impact of long-term exposure to MNZ would take into account all the days per year of exposure instead of the 8 h shift. This could explain the negative correlation observed between expected CEL values and both ETU absorbed and ETU excreted upon 8 h exposure to MNZ (r correlation values of −0.66 and −0.50, with *p* values of 0.003 and 0.034, respectively).

Even though each single worker, by wearing working clothes, was effectively shielded against exposure to MNZ, with an overall median protection percentage of 92%, the spreading group showed a slightly lower protection compared with the seedling group (89% versus 92% median percentages, respectively). Spreading procedures imply a higher exposure level; therefore, the spreading group used PPE with higher protection capacity compared with the seedling group. Nevertheless, the level of protection was lower for them. Hence, to explain this discrepancy, it would be important to verify the actual correct adherence to operating procedures according to employer indications, thereby limiting issues possibly associated with scarce personal hygiene compliance in the workplace [55].

Oxidative stress determines disturbances in cellular metabolism resulting in the formation of permanent changes caused by the oxidation of lipids, proteins, and DNA, as suggested by several studies [56]. In particular, the oxidative biomarkers chosen for this analysis, although nonspecific, have been widely linked to the toxicity of pesticides [31,57]. Following an 8 h day shift exposure to MNZ, blood levels of AOPP, ROM, and BAP biomarkers were measured and compared with those observed in non-exposed indoor CTRL workers (Figure 5). In the presence of oxidative stress conditions, plasma proteins, mainly albumin and fibrinogen, may be highly oxidized, and the oxidation level can be quantified in terms of oxidation products or AOPPs, first identified by Witko-Sarsat et al. as efficient oxidative stress biomarkers in uraemic patients [58]. Further studies confirmed AOPP concentrations as inflammatory biomarkers upon exposure to pesticides [50]. Accordingly, circulating free radical levels may also augment upon pesticide exposure, which is measured with ROM detection in serum [50]. Specifically, ROM concentrations higher than 320 CARR-U might support the presence of an oxidative-stress-related condition [59].

The results demonstrated that MNZ-exposed workers had significantly higher serum concentrations of both AOPP and ROM 3.2- and 1.6-fold higher than median values measured in CTRL samples, respectively. In particular, regarding AOPP serum concentrations, when subjects were stratified based on their job, it was found that the spreading group had a median value significantly higher than the seedling group (36.2 versus 18.2 µmol/L, respectively, with a *p* value of 0.028), demonstrating that AOPP serum readouts might be a suitable biomarker of exposure to MNZ. Although ROM concentrations in serum were not significantly different between the two groups of workers, it was possible to observe that 15 out of 19 farmers, corresponding to 79% of the whole population under study, had ROM blood values higher than 320 CARR-U, suggesting the presence of significant oxidative stress upon 8 h MNZ exposure.

Several studies have evaluated the increase in ROM in correlation with a decrease in BAP during oxidative stress conditions, including upon pesticide exposure [50,60]. In this study, in association with the increased ROM concentration, a significant decrease in the overall body antioxidant capacity, expressed as BAP concentration (µmol/L), was observed, which was found to be significantly lower in MNZ-exposed workers compared with non-exposed CTRL workers. Interestingly, the observed Pearson’s correlation coefficient between the ROM and BAP levels in MNZ-exposed subjects was positive, although not significant, possibly given the small number of subjects included in this study (r = 0.33, *p* = 0.161).

Long-term effects of MNZ exposure may include mutagenic and carcinogenic risks, as well as reprotoxicity and teratogenicity [28,61]. Importantly, the Fisher’s contingency analysis of AOPP blood concentration evidenced that, among the biomarkers analyzed, AOPP assessments may have a predictive value. In other terms, towards a personalized medical approach, AOPP measurements may be useful to identify a specific subgroup of exposed workers who could potentially develop cellular damage following exposure to MNZ, thus being at higher risk for MNZ-linked chronic pathologies compared with the whole population.

## 5. Conclusions

Despite the small sample of farmers enrolled, the obtained results confirm that the measurement of ETU levels in pads, as well as in urine, enables assessments of the risk associated with MNZ exposure. Our results evidence once more the necessity of a health-based occupational exposure limit for ETU indicated by an official agency. 

Although the study design cannot support a causal relationship between exposure to MNZ and oxidative-stress-related health effects, such biomarkers, especially AOPP, were demonstrated to be potentially valuable predictors, suggestive of increased susceptibility to chronic pathologies and cancer. For this reason, they could represent important screening tools for occupational physicians to stratify workers based on assessments of oxidative damage derived from pesticide exposure. Additional preventive and protection measures may thus be implemented for more susceptible subjects in countries where MNZ and ETU are still produced and used.

The novelty of our approach consisted in pairing the evaluation of risk associated with MNZ exposure with the biomonitoring of oxidative stress biomarkers (especially AOPP serum concentration), which might help to better prevent possible health consequences due to pesticide exposure in susceptible workers. The data collected in this study indicate that prevention and protective measures were adequate; however, the obtained results suggest that stricter control of the effective adherence to operational procedures would be desirable. Importantly, these ad hoc measurements evidenced that tiered analyses conducted in greenhouse farmers might be extended to other exposure backgrounds. Pesticide studies evolve at a fast pace; therefore, comparable future analyses will help to determine temporal trends and pivotal risk evaluations, increasing the support for public policies aimed to further reduce the use of potentially harmful pesticides.

## Figures and Tables

**Table 1:** Characteristics of the workers enrolled in the study and relative chronic exposure levels.

	All (N = 19)	Spreading (N = 2)	Seedling (N = 17)	*p* Value
Males (N, %)	2 (11%)	2 (100%)	0	na
Females (N, %)	17 (89%)	0	2 (100%)	na
Age (years)	44 (21–69)	37 (31–42)	45 (21–69)	*0.333*
Height (m)	1.63 (1.50–1.77)	1.72 (1.67–1.77)	1.58 (1.50–1.75)	*0.078*
Weight (kg)	65 (47–106)	83 (80–85)	65 (47–106)	*0.245*
BMI (kg/m^2^)	24.7 (17.9–38.9)	27.9 (27.1–28.7)	24.1 (17.9–38.9)	*0.729*
BSA (m^2^)	1.72 (1.40–2.20)	1.99 (1.93–2.04)	1.71 (1.40–2.20)	*0.125*
Working seniority (years)	6 (3–19)	8 (5–10)	6 (3–19)	*0.994*
Exposure duration (days per year)	220 (24–220)	45 (24–66)	220	*<0.0001*
Chronic exposure levels (au)	1.61 (1.01–2.47)	1.23 (1.01–1.45)	1.61 (1.32–1.47)	*0.039*

BMI, body mass index; BSA, body surface area; na, not associated; au, arbitrary units.

**Table 2:** Lifestyle habits and health assessment of the workers enrolled in the study.

Activity	No	Yes
Cigarette smoking	11 (58%)	8 (42%)
Alcohol consumption	19 (100%)	0
Daily coffee/tea use	4 (21%)	15 (79%)
Daily vegetable consumption	0	19 (100%)
Daily fruit consumption	4 (21%)	15 (79%)
Symptoms possibly related to exposure (last 12 months)	14 (74%)	5 (26%) *
Occurring pathologies	13 (68%)	6 (32%) **
Medication usage in the last 30 days	10 (53%)	9 (47%) ***

* Skin rash/erythema localized to the face (N = 3), arms (N = 1), and body (N = 1); ** Skin erythema or dermatitis (N = 2), asthma (N = 1), and thyroid dysfunction (N = 3); *** anti-inflammatory and/or antibiotic and/or anti-histaminic (N = 5), thyroid hormone (N = 3), and contraceptive (N = 1).

**Table 3:** ETU exposure assessment results from pads and urine measurements.

	All (N = 19)	Spreading (N = 2)	Seedling (N = 17)	*p* Value
PSE (mg)	0.90 (0.76–3.65)	3.49 (3.33–3.65)	0.87 (0.76–1.16)	*<0.0001*
ASE (mg)	0.07 (0.06–0.44)	0.42 (0.40–0.44)	0.07 (0.06–0.10)	*<0.0001*
Protection percentage (%)	92 (89–93)	89	92 (92–93)	*<0.0001*
ETU absorbed (µg/kg bw)	0.31 (1.44–0.24)	1.33 (1.23–1.44)	0.30 (0.24–0.38)	*<0.0001*
Risk ETU_pads_ (%)	15 (12–72)	67 (62–72)	15 (12–19)	*<0.0001*
ETU_urine_ (µg/g creatinine)	8.0 (0.5–203.8)	171.6 (139.5–203.8)	6.3 (0.5–79.3)	*<0.0001*
ETU_urine_ above 2.5 µg/g creatinine (N, %)	14 (74%)	2 (100%)	12 (71%)	na *
Risk ETU_urine_ (%)	2.8 (0.1–59.9)	51.8 (43.6–59.9)	2.5 (0.1–30.5)	*<0.001*

* na, not associated.

## Data Availability

Not applicable.

## Supplementary Materials

The following supporting information can be downloaded at: www.mdpi.com/article/10.3390/ijerph191710486/s1, Table S1: Electrospray ionization interface (ESI) voltage settings. Table S2: Mass spectra (MS) detection and selected ions. Table S3: Limit of detection (LOD) and limit of quantification (LOQ) for pad and urine samples. Table S4: Intra-day accuracy and precision of the assay at three different concentration levels. Table S5: Inter-day precision and accuracy of the assay at three different concentration levels.
