# Supplementary material for: Assessment of Mancozeb Exposure, Absorbed Dose, and Oxidative Damage in Greenhouse Farmers
Source: Int J Environ Res Public Health. 2022 Aug 23;19(17):10486. doi: 10.3390/ijerph191710486 (PMC9518406; doi:10.3390/ijerph191710486)
Supplement: Supplementary file 1 [file ijerph-19-10486-s001.zip › ijerph-1861331-supplementary.pdf]

Supplementary Material

ASSESSMENT OF MANCOZEB EXPOSURE, ABSORBED DOSE AND OXIDATIVE DAMAGE IN GREENHOUSE FARMERS

Chiara Costa[1], Michele Teodoro[2], Federica Giambò[2], Stefania Catania[2], Silvia Vivarelli[2*], Concettina Fenga[2]

Supplementary Table S1. Electrospray ionization interface (ESI) voltage settings.

**MS detection parameters**	
Capillary (kV)	0.3
Cone (V)	31
Extractor (V)	3
RF Lens (V)	0.4
Source temperature (◦C)	130
Desolvation temperature (◦C)	450
Cone gas flow (l/h)	51
Desolvation gas flow (l/h)	800

Supplementary Table S2. Mass spectra (MS) detection and selected ion.

**Selected ion**	**Parents (m/z)**	**Daughter (m/z)**	**Identifier (m/z)**	**Collision (V)**	**Retention time (min)**
		**44.1**		22	
ETU	102.9	59.9	44.1	31	2.045
		102.9		11	
		48.1		22	
[$^2H_4$]-ETU	106,9	**63.9**	63.9	22	1.95
		106.9		12	

Note: Bold characters are used for daughter ions quantifiers

Supplementary Table S3. Limit of detection (LOD) and limit of quantification (LOQ) for pad and urine samples.

**Matrix**	**LOD (μg/l) (n=10)**	**Accuracy (% ± SD)**	**Precision %CV**	**LOQ (μg/l) (n=10)**	**Accuracy (% ± SD)**	**Precision %CV**
Water (pads)	0.2078±0.0135	103.90±0.065	20.40	0.2497±0.0039	99.88±0.016	5.65
Urine	0.2086±0.0085	101.80±0.0229	6.8	0.2545±0.0058	101.80±0.0229	6.8

SD, Standard deviation; CV, Coefficient of variation

Supplementary Table S4. Intraday accuracy and precision of the assay at three different concentration levels.

Matrix	n	Concentration (µg/l)	ETU Concentration Mean obtained (µg/l)	Accuracy (% ± SD)	Precision %CV
Water (pads)	15	0.5	0.507	101.48±1.07	5.58
		5	5.091	101.41±1.51	5.42
		50	50.433	100.87±0.93	4.16
Urine	15	0.5	0.544	108.76±2.82	8.62
		5	5.122	102.45±1.51	5.38
		50	50.464	100.93±0.93	4.16

Supplementary Table S5. Interday precision and accuracy of the assay at three different concentration levels.

Matrix	n	Concentration (µg/l)	Mean obtained ETU Concentration (ng/ml)	Accuracy (% ± SD)	Precision %CV
Water (pads)	18	0.5	0.510	102.09±2.65	6.50
		5	4.956	99.12±3.52	9.03
		50	50.085	100.17±4.30	9.34
Urine	18	0.5	0.508	101.63±2.76	6.14
		5	4.997	99.94±3.58	9.25
		50	50.917	101.83±2.07	2.84
